# Pharmacological potential of endocannabinoid and endocannabinoid-like compounds in protecting intestinal structure and metabolism under high-fat conditions

**DOI:** 10.3389/fphar.2025.1567543

**Published:** 2025-05-22

**Authors:** Francesco Vari, Ilaria Serra, Marzia Friuli, Viviana Cavallo, Nicola Gammaldi, Daniele Vergara, Michel Salzet, Anna M. Giudetti

**Affiliations:** ^1^ Department of Physiology and Pharmacology “V. Erspamer”, Sapienza University of Rome, Rome, Italy; ^2^ Department of Biological and Environmental Sciences and Technologies (DiSTeBA), University of Salento, Lecce, Italy; ^3^ University of Lille, Inserm, CHU Lille, U-1192 - Laboratoire Protéomique Réponse Inflammatoire Spectrométrie de Masse - PRISM, Lille, France

**Keywords:** endocannabinoid, gut health, high-fat diet, metabolic disorders, intestinal metabolism, oleoylethanolamide

## Abstract

The intestine plays a crucial role in nutrient absorption, digestion, and regulation of metabolic processes. Intestinal structure and functions are influenced by several factors, with dietary composition being one of the most significant. Diets rich in various types of fats, including saturated, monounsaturated, and polyunsaturated fats, have distinct effects on intestinal cell metabolism and overall intestinal health. High consumption of saturated fats, frequently found in animal products, has been associated with inflammation, altered gut microbiota composition, and impaired intestinal barrier function, with potential consequences such as metabolic disorders, obesity, and insulin resistance. In contrast, monounsaturated fats, found in foods such as olive oil and avocado, promote intestinal cell integrity, reducing inflammation and supporting a healthier microbiome. Polyunsaturated fatty acids, especially omega-3 fatty acids, have shown anti-inflammatory effects and may improve the function and adaptability of intestinal cells, promoting better nutrient absorption and immune regulation. Recent evidence suggests that endocannabinoids and endocannabinoid-like compounds, such as oleoylethanolamide have a protective effect on the function and structure of the intestine. These endocannabinoid pathways modulating compounds can act on receptors in the intestinal epithelium, improving the intestinal barrier and counteracting inflammation, facilitating a more favorable environment for intestinal health. Understanding how different fats influence intestinal metabolism and the protective role of endocannabinoids and endocannabinoid-like compounds is essential to developing dietary strategies to improve intestinal health and prevent diet-related diseases. This review explores the impact of high fats on intestinal metabolism and the main role of endocannabinoids and endocannabinoid-like compounds on these effects.

## Introduction

The human intestinal system is a highly intricate organ in terms of physiological functions and structural organization. It is essential for digestion, nutrient absorption, immune defense, and maintaining symbiotic relationships with the gut microbiota (Hickey et al., 2023). Comprising the small and large intestines, the gut is uniquely structured to perform these multifaceted roles through specialized anatomical formations, a diverse cellular composition, and a dynamic biochemical environment. Recent advancements in research techniques have unveiled the intricate organization and functioning of the gut, illustrating how its structural complexities support physiological functions critical to human health (Hickey et al., 2023).

The small intestine, consisting of the distinct regions of the duodenum, jejunum, and ileum, has a highly folded mucosal surface with villi and microvilli on epithelial cells. This extensive surface area facilitates the efficient absorption of nutrients, electrolytes, and water, including sugars, monovalent ions, and amino acids (Nigam et al., 2019). The lamina propria, a connective tissue layer within each villus, contains blood and lymphatic vessels that transport absorbed nutrients and lipids to the body (Hickey et al., 2023; Nigam et al., 2019).

The large intestine is divided into the ascending, transverse, descending, and sigmoid sections, which include the cecum, colon, and rectum, facilitating feces formation and excretion. Lacking villi, it has deep crypts with cells that produce mucus, aiding in lubrication. Its main function is absorbing water, electrolytes, vitamins, and the anaerobic fermentation of dietary fibers. Paracellular or transcellular pathways regulate the movement of solutes across the epithelium, with active transport for nutrient and electrolyte absorption (Hickey et al., 2023; Nigam et al., 2019). Most digestion and absorption processes occur in the duodenum, jejunum, and ileum, where nutrients are absorbed into capillaries and lymphatic vessels. Unabsorbed material moves into the colon, where water is absorbed, and feces are formed and stored (Hickey et al., 2023).

High-fat diets (HFDs) are known to have a significant impact on gut health, contributing to various metabolic disorders such as dysbiosis, increased intestinal permeability, and inflammation, all of which impair the gut’s ability to regulate nutrient absorption and protect against harmful pathogens. These alterations in intestinal function are linked to systemic metabolic dysfunction, including obesity and insulin resistance (Giudetti et al., 2021). Recent research has highlighted the potential therapeutic role of endocannabinoids and endocannabinoid-like compounds in mitigating the negative effects of HFDs. Among endocannabinoid-like compounds, oleoylethanolamide (OEA) has garnered significant interest due to its beneficial effects on various physiological functions, including the regulation of dietary fat intake, energy balance, and intestinal motility, as well as its influence on eating behavior (Tutunchi et al., 2020). In the small intestine, particularly in the duodenum and jejunum, OEA levels fluctuate in response to nutritional status, decreasing during periods of starvation and rising upon refeeding (Bowen et al., 2017). Recent research has demonstrated that administering OEA to rodents can provide protection against inflammation and alter the composition of the intestinal microbiota (De Filippo et al., 2023). This review aims to examine the effects of HFDs on intestinal barrier function and structure, discussing how endocannabinoids and endocannabinoid-like compounds, particularly OEA, may safeguard the gut from damage induced by HFDs.

## Intestinal barrier: structure, properties, and role in gut health

The intestinal barrier is a multifunctional interface critical for nutrient absorption and immune defense. It comprises epithelial cells, mucosa, immune components, and microbiota, all of which maintain a selectively permeable structure that allows the passage of essential nutrients and water while restricting pathogenic organisms and toxins (Shalon et al., 2023; Di Tommaso et al., 2021). Recent advancements in single-cell RNA sequencing have revealed the cellular diversity of the gut (Hickey et al., 2023). The intestinal epithelium consists of five main cell types: enterocytes, goblet cells, enteroendocrine cells, Paneth cells, and M cells, derived from stem cells in the crypts. Enterocytes absorb nutrients *via* specific transporters, goblet cells secrete mucus, Paneth cells produce antimicrobial peptides (AMPs), and enteroendocrine cells release hormones regulating digestion and appetite (Di Tommaso et al., 2021). The mucus layer, produced by goblet cells, primarily consisting of mucin proteins, represents the first layer of defense. Consistent with their functions, mucins are classified as transmembrane and secretory. Transmembrane mucins include MUC1, MUC3A/B, MUC4, MUC12, MUC13, MUC15, MUC17, MUC20, and MUC21 that, thanks to their transmembrane structure, also participate in signal transduction. The secretory mucins are classified as gel-forming and non-gel-forming mucins. The gel-forming mucins include MUC2, MUC5AC, MUC5B, MUC6, and MUC19, and among them, MUC2 is the most typical gel-forming mucin expressed in the jejunum, ileum, and colon (Qiao et al., 2025). The mucus layer physically separates microbiota from the epithelial cells, reducing direct interactions with pathogens. Beneath this, the epithelial layer supports barrier integrity by producing AMPs and aiding nutrient transport (Di Tommaso et al., 2021; Birchenough et al., 2023). AMPs are small, naturally occurring proteins that play a crucial role in the innate immune response by attracting immune cells to the site of infection, promoting inflammation, and enhancing the activity of other immune components (Gagandeep et al., 2024). Thus, AMPs play an essential role in the first line of defense against pathogens, including bacteria, viruses, fungi, and parasites. The primary structure of AMPs exhibits considerable variability. Larger AMPs, composed of 100 amino acids or more, often share similarities with lytic enzymes, nutrient-binding proteins, or proteins with specific binding sites for microbial macromolecules. Conversely, most AMPs are smaller and primarily involved in disrupting the structural integrity or functionality of microbial cell membranes. They can also directly inhibit specific adenosine triphosphate (ATP)-dependent enzymes through their interaction with ATP. In humans, the principal classes of AMPs are cathelicidins and defensins, with defensins further classified into alpha (α) and beta (β) types (Yang et al., 2025). Additionally, certain AMPs contribute to tissue repair and regeneration by facilitating cell migration and proliferation, which are crucial for effective wound healing (Duarte-Mata and Salinas-Carmona, 2023). Tight junctions (TJs), composed of proteins such as claudins, occludins, and Zonula Occludens (ZO), seal the space between adjacent enterocytes, preventing the passage of harmful pathogens and toxins into the bloodstream. Adherens junctions (AJs), formed by cadherins and linked to the actin cytoskeleton, provide mechanical strength and regulate cell-cell adhesion. Desmosomes offer additional structural support by connecting intermediate filaments between cells, ensuring tissue resilience under mechanical stress (Shalon et al., 2023; Di Tommaso et al., 2021). Gap junctions (GJ), made of connexins, allow for the direct transfer of ions and small molecules between cells, enabling coordinated cellular activity. Together, these junctions ensure the proper functioning of the intestinal barrier, maintaining a selective permeability essential for digestion and immune defense (Di Tommaso et al., 2021).

Due to the impermeability of the intestinal epithelium to hydrophilic solutes, specific transporters mediate nutrient passage *via* transcellular and paracellular pathways. The transcellular pathway involves active transport, endocytosis, and nutrient-specific transporters, while the paracellular pathway allows ions and hydrophilic molecules through junctional complexes. Under pathological conditions, increased epithelial permeability allows the translocation of harmful agents, such as luminal antigens and microbial toxins, which can activate afferent nerves and lead to visceral hypersensitivity. This enhanced permeability is associated with diseases like inflammatory bowel disease (IBD), celiac disease, and irritable bowel syndrome (IBS) (Shalon et al., 2023).

The function of the intestine is also closely related to neural and immune components (Greenwood-Van Meerveld et al., 2017). The GI tract is regulated by the enteric nervous system (ENS), a complex, autonomous network that controls digestive functions independently of the central nervous system. The ENS comprises sensory neurons, motor neurons, and interneurons that coordinate muscular movements and the intestine’s secretory and absorptive activities, involving the autonomic nervous system (ANS). The ANS contributes to GI regulation by transmitting sensory signals to the brain *via* parasympathetic and sympathetic pathways. This bidirectional gut-brain connection influences not only motility and secretions but also visceral sensitivity and pain perception (Greenwood-Van Meerveld et al., 2017).

## Intestinal microbiota

The intestinal microbiota, also known as gut microbiota, refers to the vast community of microorganisms, including bacteria, viruses, fungi, and archaea, which reside in the GI tract, particularly the intestines. The intestinal microbiota influences ENS development and interacts with immune and epithelial cells by producing metabolites that support colon cell health, the immune system, barrier function, metabolic processes, and gut-brain communication (Shalon et al., 2023; Greenwood-Van Meerveld et al., 2017; Fukasawa et al., 2025).

Beneficial bacteria in the gut, including *Bifidobacteria and Firmicutes*, ferment dietary fibers, especially non-digestible carbohydrates such as cellulose, pectin, and resistant starch, to produce short-chain fatty acids (SCFAs) (Shalon et al., 2023). SCFAs, which include butyrate, propionate, and acetate, produced in a roughly 3:1:1 ratio, play a significant role in maintaining gut health and supporting various physiological processes (O'Riordan et al., 2022). The amounts of SCFAs vary significantly among fecal samples, being influenced by individual lifestyle and health conditions (Huda-Faujan et al., 2010; Zhang et al., 2025). Acetate is produced primarily by bacteria such as *Bacteroides*. Propionate, primarily produced by *Firmicutes*, is important for regulating lipid metabolism. Butyrate, produced by specific bacteria such as *Faecalibacterium prausnitzii* and *Butyrivibrio fibrisolvens*, is a primary energy source for colonocytes (cells lining the colon) and has anti-inflammatory properties. Bacterial metabolites also encompass valeric acid, which is produced through the gut microbiota fermentation of dietary fibers, as well as isovalerate and isobutyrate, both of which are branched SCFA derived from the fermentation of branched-chain amino acids that result from undigested proteins reaching the colon. Valeric acid is noted for its anti-inflammatory properties and influence on energy metabolism; it has also been studied for its effects on mood and anxiety (Xiong et al., 2022; Gharib et al., 2015). Isovaleric acid, primarily generated from the fermentation of leucine, has been linked to various effects on muscle metabolism and energy production, and it may contribute to gut health by promoting relaxation of colonic smooth muscle through the PKA pathway (Blakeney et al., 2019). Isobutyrate has been associated with several physiological effects, particularly concerning the insulin signaling pathway. Indeed, isobutyrate enhances lipid and glucose metabolism in adipocytes, which may improve insulin sensitivity (Heimann et al., 2016). SCFAs enhance TJ integrity, increase mucus production, and support anti-inflammatory responses by modulating immune cell activity (Ney et al., 2023). Additionally, specific microbial strains and metabolites influence immune reactions through the gut-brain and gut-liver axes, impacting local and systemic immunity (Di Tommaso et al., 2021; Greenwood-Van Meerveld et al., 2017).

## Exploring the endocannabinoid system: functions and implications

The endocannabinoid system (ECS) is a complex signaling network that regulates various physiological processes, including energy balance, lipid metabolism, appetite, inflammation, and neuroprotection (Piomelli, 2003; Di Marzo and Matias, 2005). ECS comprises endocannabinoids, natural compounds that resemble the cannabinoids found in cannabis, cannabinoid receptors, and enzymes involved in both the synthesis and degradation of endocannabinoids. Particularly, the degradative enzymes include fatty acid amide hydrolase (FAAH) and monoacylglycerol lipase (MAGL) that tightly regulate endocannabinoid levels to ensure proper signaling and prevent receptor overstimulation (Di Marzo and Matias, 2005). Dysregulation of FAAH and MAGL activity can lead to altered endocannabinoid signaling, contributing to conditions like chronic pain, obesity, and metabolic disorders (De Filippo et al., 2023; Cravatt et al., 2001).

There are two main types of cannabinoid receptors: CB1, which is found primarily in the brain and central nervous system, and CB2, which is found primarily in peripheral organs and the immune system. Beyond the classical CB1 and CB2 pathways, endocannabinoids also interact with transient receptor potential (TRP) channels and PPARs, further broadening their functional repertoire (Lo et al., 2005).

Anandamide (AEA), a lipid-signaling molecule belonging to the N-acylethanolamine (NAE) family, and 2-arachidonoylglycerol (2-AG) are the most extensively studied endocannabinoids. Endocannabinoid-like compounds are endogenous lipid mediators structurally related to AEA; they share several steps of synthesis and degradation with AEA but have distinct receptor-binding profiles. These molecules, including OEA, palmitoylethanolamide (PEA), stearoylethanolamide (SEA), N-linoleoylethanolamine (LEA), and N-docosahexaenoylethanolamine (DHEA), do not directly activate CB1 or CB2 receptors but modulate physiological processes through other pathways. OEA is a prominent member of the NAE family, particularly recognized for its significant role in the GI system. It is distinguished as the most potent NAE agonist for the peroxisome proliferator-activated receptor α (PPARα) through which it exerts anorectic effects (Fu et al., 2003). In addition to its appetite-suppressing properties, OEA demonstrates anti-inflammatory, neuroprotective, and analgesic effects, likely mediated by PPARα activation, although PPARα-independent mechanisms may also play a role (Suardíaz et al., 2007). Notably, OEA exhibits *in vitro* affinity for the GPR119 receptor, although its anorectic effect does not seem to rely on this receptor interaction (Im, 2021). Furthermore, OEA promotes protein kinase C (PKC)-dependent phosphorylation and activation of the TRPV1 channel, thereby contributing to the excitation of sensory nerves (Ahern, 2003). In the rat brain, OEA levels are approximately one-third that of PEA and SEA, with a significant increase observed during cerebral ischemia, suggesting a potential neuroprotective role for the NAE family through various molecular mechanisms (Mock et al., 2023).

A randomized clinical trial demonstrated that dietary supplement with PEA added with polydatin induced a markedly improved abdominal pain severity in patients with IBS (Cremon et al., 2017). Moreover, PEA can also display its bioactive effect throughout GPR119 and GPR55, although the interaction with the latter has been questioned (Im, 2021). Although it shares several bioactivities with PEA, SEA does not interact with PPARα receptors but shows affinity for the GPR119 receptor (Mock et al., 2023). SEA has anti-inflammatory, anorectic, and neuroprotective effects (Winiarska-Mieczan et al., 2023). While SEA has received less attention, emerging evidence suggests it may contribute to cellular homeostasis and metabolic regulation (Di Marzo et al., 1998).

LEA has not been as extensively studied as other members of the NAE family despite sharing similar bioactivities with OEA and PEA. LEA reduced food intake in a manner dependent on PPARα activation, like the effects of OEA and PEA (Mock et al., 2023). Given its high intestinal concentration, it is suggested that LEA’s anorectic effects may also involve GPR119, where it exhibits comparable activity to OEA, although this remains to be validated (Mock et al., 2023).

Over the past decade, DHEA has emerged as a new member of the NAE family with distinct neuronal properties (Kim and Spector, 2018), leading to its designation as synaptamide (Kim et al., 2011). DHEA exhibits nanomolar affinity for GPR110, a G protein-coupled receptor highly expressed in the hippocampus (Lee et al., 2016) and promotes neurite outgrowth and synapse formation in wild-type neurons throughout GPR110 activation (Huang et al., 2020; Qu et al., 2022). Additionally, DHEA has GPR110-dependent anti-inflammatory effects, reducing pro-inflammatory cytokines in LPS-treated microglia and decreasing neuroinflammation in mice (Park et al., 2016; Meijerink et al., 2015; Park et al., 2019). DHEA levels in the brain are significantly higher than AEA, and its concentration correlates with brain docosahexaenoic acid levels. Limited research has explored DHEA’s physiological role in peripheral tissues (Mock et al., 2023). The endocannabinoid-like compounds often interact synergistically with endocannabinoids, amplifying or modulating ECS activity. For instance, PEA has been observed to enhance the activity of AEA by reducing FAAH-mediated degradation, a phenomenon known as the “entourage effect” (Srivastava et al., 2022).

## The role of endocannabinoids in gut structure and function

The ECS is extensively expressed throughout the GI tract, encompassing enterocytes, immune cells, and ENS (Cani et al., 2014; Galiazzo et al., 2018). This system plays a pivotal role in gut physiology, intricately modulating motility, permeability, and inflammatory responses. Importantly, various studies, primarily summarized in Table 1, highlight the dual role of cannabinoids in modulating intestinal permeability, which has significant implications for both healthy and inflammatory conditions. Under physiological conditions, the action of endocannabinoids in the GI tract is predominantly mediated by the CB1 receptor (Izzo and Sharkey, 2010). However, CB1 activation can increase epithelial permeability by reducing TJ expression, creating conditions that may promote obesity (Maccarrone et al., 2015). On the contrary, in pathological states, both CB1 and CB2 reduce abnormal GI motility and permeability (Duncan et al., 2008).

**TABLE 1:** Effects of endocannabinoids, endocannabinoid-like compounds, and cannabinoids on the intestinal barrier.

Endocannabinoid		Endocannabinoid-like		Cannabinoid
MAIN TYPES				
2-AG AEA 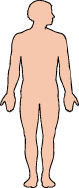		PEA DHEA LEA 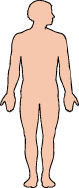 OEA SEA		THC CBD 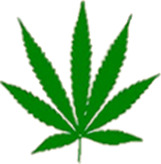
MAIN RECEPTORS				
CB1-CB2		PPARα-TRPV1-GPR119-GRPR110		CB1-CB2

Compound(s)	Model / Species	Experimental Design	Effect / Outcome	Ref.
AEA, 2-AG	Caco-2 cells	EDTA-induced TEER decrease	Exacerbated TEER decrease when applied apically	Alhamoruni et al., 2010
AEA, 2-AG	Caco-2 cells	Inflammatory condition induced by cytokines	Exacerbated TEER decrease when applied apically	Alhamoruni et al., 2012
HU210 (CB1 Agonist)	Mice	CB1 Induction	Increased permeability	Maccioni et al., 2025
	CB1 knockout mice	Immobilization and acoustic stress induced	More severe intestinal barrier disruption in CB1 knockout mice compared to wild type	Zoppi et al., 2012
AEA	Mice-T84 cells	CB2 induction	Suppress neutrophil transmigration	Szabady et al., 2018
AEA	Mice	Inflammatory condition induced	Induced AMPs and the abundance of beneficial bacteria	Sultan et al., 2021
PEA	Mice	Colitis model	Reduced inflammation	Borrelli et al., 2015
	Rats-IEC6 cells	Aging model	Aging and the reduction of CB1 expression are correlated with decreased intestinal integrity	Lee et al., 2023
SR141716A (CB1 antagonist)	Mice	CB1 antagonism	Increased gut integrity, increased beneficial gut bacteria and boosts of SCFA production	Mehrpouya-Bahrami et al., 2017
CP55940 (CB1-CB2 agonist)	Mice	CB1 induction	Restored intestinal permeability induced by high fat diet	Cuddihey et al., 2022
CBD, THC	Mice	HFD rich in cholesterol	Reduced gut microbiota disturbances, reduced intestinal inflammation	Gorelick et al., 2022
PEA	Mice	HFD	Improved gut barrier integrity and growth of beneficial microbes	Schwartz et al., 2008

Endocannabinoids (AEA and 2-AG), endocannabinoid-like compounds (OEA and PEA), and cannabinoids THC (delta-9-tetrahydrocannabinol) and CBD (cannabidiol) modulate intestinal health through multiple mechanisms. These include enhancing intestinal barrier integrity, regulating gut microbiota composition, modulating inflammatory responses, and influencing metabolic pathways. Experimental models reveal receptor-specific effects involving cannabinoid receptors, PPARα, TRPV1, and others, highlighting their therapeutic potential in gastrointestinal and metabolic disorders.

In Caco-2 cells, AEA and 2-AG exacerbated apically EDTA-induced TEER decrease but facilitated a concentration-dependent recovery of basolateral TEER. These effects were inhibited by the CB1 receptor antagonist AM251 and the TRPV1 antagonist capsazepine in the case of AEA (Alhamoruni et al., 2010). Apical application of AEA and 2-AG worsened hyperpermeability in Caco-2 cells exposed to inflammatory cytokines throughout CB1, and co-application of AEA with a FAAH inhibitor, or 2-AG with a MAGL inhibitor, further decreased TEER (Alhamoruni et al., 2012). FAAH and MAGL inhibitors, such as URB597 and JZL184, caused concentration-dependent drops in TEER in Caco-2 cells. Furthermore, URB597 and JZL184 worsened TEER reductions in cells exposed to inflammatory cytokines and hypoxia. The effects were absent in CB1 knockdown Caco-2 cells (Karwad et al., 2017a). Alcohol-induced increased intestinal permeability is reversed by inhibiting the CB1 receptor with selective CB1 receptor antagonists (Maccioni et al., 2025).

*In vivo* research reinforced ECS involvement, particularly the CB1 receptor, in the control of intestinal permeability. Chronic stimulation of the CB1 receptor with the agonist HU210 in wild-type mice led to an increased permeability (Muccioli et al., 2010). Conversely, CB1 knockout mice, compared to wild-type mice, experienced more severe intestinal barrier disruption after exposure to immobilization and acoustic stress (Zoppi et al., 2012).

The ECS has an intriguing connection with the gut microbiota. Germ-free mice showed increased CB1 receptor and endocannabinoid levels, effects that were reversed by the reconstitution of gut microflora (Manca et al., 2020). Antibiotics also affect the ECS, increasing CB2 receptor expression, especially under stress conditions (Guida et al., 2018). Probiotics *Lactobacillus acidophilus* can enhance CB2 receptors and reduce mice’s visceral hypersensitivity (Aguilera et al., 2013; Vijay et al., 2021). Baseline levels of NAEs were positively associated with gut bacterial diversity and beneficial SCFA-producing species like *Bifidobacterium* and *Faecalibacterium*. Increased AEA correlated with elevated butyrate levels, increased AEA and PEA, and reduced inflammatory cytokines. On the other hand, 2-AG and OEA were associated with higher levels of the anti-inflammatory cytokine IL-10, highlighting the ECS’s role in mediating anti-inflammatory actions *via* the gut microbiota (Vijay et al., 2021). In this context, the use of prebiotics or probiotics for improving the tone of the ECS, and endocannabinoids such as PEA, to restore the normal intestinal microbiota has been reported to ameliorate GI dysfunctions (Turco et al., 2023; Szabady et al., 2018).

The ECS and the CB2 receptor play an essential role in intestinal inflammation (Szabady et al., 2018). AEA has been shown to suppress neutrophil migration through the CB2 receptor, although 2-AG, another CB2 receptor agonist, does not show the same effect (Szabady et al., 2018).

Studies in mice have shown that CB2 receptor absence leads to more severe disease and higher intestinal neutrophil accumulation, suggesting that CB2 activation helps prevent excessive immune responses (Szabady et al., 2018). Pharmacological activation of CB2 or the elevation of endocannabinoid levels through FAAH inhibition has been shown to protect against an experimental mouse model of colitis (Andrzejak et al., 2011).

AEA can also reverse the inflammation-induced increase in intestinal pathogenic bacteria, such as *Pseudomonas*, by inducing several AMPs, and can increase the abundance of beneficial bacteria that produce the SCFA butyrate. Thus, increasing endogenous AEA through FAAH inhibitors has been reported as an effective treatment for inflammation-based diseases (Sultan et al., 2021). PEA has demonstrated significant benefits in reducing inflammation in colitis models (Borrelli et al., 2015). Beyond CB2 receptors, PEA also influences enteric glial cells, which are critical for regulating inflammation and maintaining the integrity of the intestinal lining (Zeisel et al., 2018). Furthermore, the regulation of the level of PEA and AEA through enzymes such as FAAH and NAAA offers a possible strategy to control inflammation in the gut and improve mucosal integrity (Alhouayek et al., 2015). Moreover, OEA treatment in rodents protects against inflammatory events and changes the intestinal microbiota composition (De Filippo et al., 2023). Results demonstrated that CB1 signaling may be a useful strategy to reduce intestinal permeability in aging-related gut inflammatory conditions (Lee et al., 2023).

Based on emerging data, endocannabinoids may serve as valuable biomarkers for intestinal diseases such as IBS, IBD, and colorectal cancer (CRC) (Cuddihey et al., 2022a). The observed elevations in plasma levels of AEA and OEA in patients with ulcerative colitis (UC) and Crohn’s disease (CD), along with the increased levels of 2-AG in CD and CRC patients, highlight their potential role in disease pathology. Furthermore, the upregulation of the 2-AG synthesizing enzyme diacylglycerol lipase (DAGL) alpha in intestinal biopsies from CD patients, coupled with the altered expression of GPR119, underscores the involvement of ECS in these conditions (Grill et al., 2019). The ability of NAEs to promote the expansion of Enterobacteriaceae, a hallmark of inflammatory bowel disease, suggests that these compounds not only reflect disease status but may also contribute to disease progression. Given these findings, endocannabinoids could be utilized as biomarkers to diagnose and monitor intestinal diseases, provide insights into disease mechanisms, and potentially guide therapeutic strategies (Fornelos et al., 2020).

## OEA synthesis and metabolism

**FIGURE 1:**
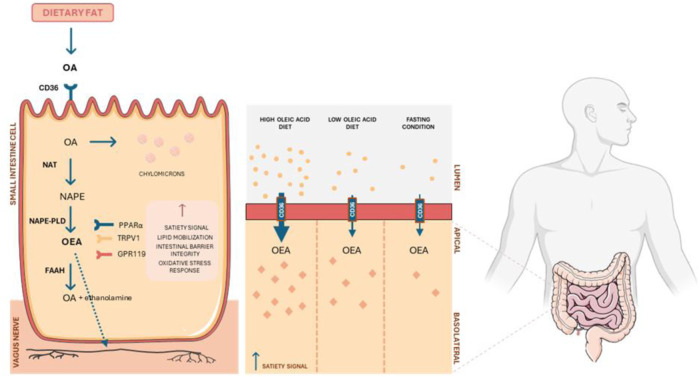
Mechanism of OEA production in small intestinal cells and its role in metabolic regulation. Dietary fat containing oleic acid (OA) is metabolized in enterocytes, producing NAPE and subsequent synthesis of OEA *via* NAPE-PLD enzymatic activity. OEA interacts with receptors such as PPARα, TRPV1, and GPR119, modulating satiety signals, lipid mobilization, intestinal barrier integrity, and oxidative stress response. FAAH breaks down OEA into OA and ethanolamine. The diagram also compares OEA production under diets with high and low oleic acid content, highlighting its physiological impact through vagus nerve activation.

OEA, an endocannabinoid-like compound, has garnered attention as a potential therapeutic agent for different diseases due to its unique mechanisms of action, distinct from those of traditional endocannabinoids. OEA synthesis occurs in the proximal small intestine in response to dietary fat intake through a two-step process (Figure 1) (Igarashi et al., 2021). First, the enzyme N-acyl transferase (NAT) catalyzes the bonding between the free amino group of phosphatidylethanolamine (PE) and the oleoyl group in sn-1-oleoyl-phosphatidylcholine. This reaction forms N-acylphosphatidylethanolamine (NAPE). In the second step, NAPE is hydrolyzed by N-acylphosphatidyl-ethanolamine-specific phospholipase D (NAPE-PLD), producing phosphatidic acid and OEA. The biosynthesis of OEA and other bioactive lipid amides is modulated by bile acids (Igarashi et al., 2021; Fu et al., 2007).

During feeding, OEA levels in the duodenal and jejunal mucosa increase due to enhanced activity of NAPE-PLD, while fasting promotes degradation through increased FAAH activity (Fu et al., 2007; Terrazzino et al., 2004). Localization studies confirm that OEA biosynthesis and hydrolysis occur in intestinal epithelial and lamina propria cells. Unlike classical satiety peptides, OEA acts as a paracrine signal within the gut to extend postprandial intervals, underscoring its therapeutic potential for obesity and metabolic disorders (Diao et al., 2022).

OEA does not activate CB1 or CB2 receptors. Instead, it primarily activates PPARα, regulating genes critical to fat absorption and fatty acid metabolism (Iannotti and Vitale, 2021). Interestingly, activation of PPARα itself also contributes to this process by modulating the expression of satiety-associated proteins, including apolipoprotein A-IV, enhancing its role in appetite control (Tso and Liu, 2004). In addition to PPARα-mediated effects, OEA interacts with GPR119 and TRPV1, which contribute to other physiological processes, including energy homeostasis, inflammation, and sensory regulation (Diao et al., 2022). Furthermore, recent research has revealed that OEA functions as an endogenous ligand for hypoxia-inducible factor 3-alpha (HIF-3α). This novel role links OEA to the regulation of lipid metabolism and obesity while integrating oxygen-dependent pathways into its metabolic functions (Diao et al., 2022).

The anorexigenic effects of exogenously administered OEA are accompanied by activation of specific brain regions, including the hypothalamic paraventricular nucleus and the brainstem, which are associated with satiety control (Romano et al., 2023). Notably, OEA does not suppress food intake when administered intracerebrally, and its effects are blocked by capsaicin-induced desensitization of peripheral sensory fibers. However, it has been demonstrated that vagal afferent fibers are not strictly necessary for both behavioral and neurochemical effects of OEA (Romano et al., 2023; Karimian Azari et al., 2014). Within a few minutes after its intraperitoneal administration, an increased concentration of intact OEA is observed in different brain areas, with associated inhibition of food intake. These data support the hypothesis that OEA, probably through circulation, rapidly reaches the brain and inhibits eating by acting directly on selected brain nuclei (Romano et al., 2023). These findings establish OEA as a peripheral lipid mediator regulating feeding behavior (Rodríguez de Fonseca et al., 2001).

Intraperitoneal injection of OEA ensures its efficacy by bypassing GI degradation (Tso and Liu, 2004). However, a study by Nielsen et al. demonstrated that an oral dose of 10 mg/kg OEA in 24-hour-starved rats is nearly as effective as intraperitoneal administration. The study observed a significant reduction in radiolabeled [^3^H]OEA during its transit from the stomach to the intestine, primarily due to extensive enzymatic hydrolysis by FAAH. Despite this, 0.48% of the administered dose remained intact in the intestinal tissue after 90 min, effectively inhibiting food intake by 15.5%. This retained amount, 11 times higher than the endogenous level of 0.354 nmol/g, accounts for its anorectic efficacy. Importantly, the study confirmed that OEA’s anorectic effects are mediated by the intact molecule rather than its radiolabeled metabolites, oleate, and ethanolamine. Tests with oral ethanolamine (1.88 mg/kg) and oleate (8.68 mg/kg) independently showed no significant impact on food intake, emphasizing that the anorectic effects are specific to intact OEA administration. The finding that this simple and naturally occurring compound holds potential for oral use is highly advantageous for anti-obesity medicine development (Rodríguez de Fonseca et al., 2001; Nielsen et al., 2004).

Drug delivery involves methods and technologies designed to transport therapeutic compounds to their target sites in the body, ensuring maximum efficacy and minimal side effects (De Jong and Borm, 2008). A study presents PEGylated liposomes encapsulating OEA to enhance its solubility and bioavailability for stroke therapy. Intravenously administered liposomes demonstrated controlled release, reduced neuronal apoptosis, and attenuated inflammation in ischemic stroke models (Wu and Yang, 2022). OEA, contrary to other satiety agents like cholecystokinin, not only suppresses meal size but also prolongs the intervals between meals, providing a unique mechanism for appetite regulation (Terrazzino et al., 2004). Unlike CB1 receptor antagonists, which have been associated with severe psychiatric side effects, OEA offers a safer alternative with minimal adverse effects for anti-obesity therapies (Romano et al., 2014).

## *In vitro* and *in* vivo approaches to investigate the effects of HFD

HFD formulations are frequently employed in experimental research to investigate how elevated lipids influence metabolic processes, cellular function, and the overall health of biological systems (Greenwood-Van Meerveld et al., 2017). The duration of feeding and the types of fats used, whether saturated, unsaturated, or a combination, are critical factors influencing experimental outcomes (Di Tommaso et al., 2021). The HFDs used *in vivo* studies vary significantly in macronutrient composition, particularly in the ratio of saturated fatty acids (SFA) to unsaturated fatty acids (Recena et al., 2019). These formulations typically provide 40%–60% of total calories from fats (Nguyen et al., 2017). HFD formulations rich in SFA, reproducing a Western diet model, combine fats from sources like lard or palm oil (Xiong et al., 2022). Short-term exposure to these diets (1–7 days) primarily focuses on rapid metabolic changes, including intestinal proliferation, insulin resistance, and lipid absorption (Shalon et al., 2023; Keles et al., 2025), while medium-term studies (8–12 weeks) evaluate systemic effects, such as gut barrier disruption, inflammation, and the onset of non-alcoholic fatty liver disease (NAFLD) (Jiang and Miao, 2023). Long-term protocols (6–12 months) assess chronic adaptations, such as obesity, tumorigenesis, and immune dysregulation (Shalon et al., 2023). For example, mice fed 60% fat diets for 9–14 months showed significant increases in intestinal stem cell proliferation and tumor development (Birchenough et al., 2023).

*In vitro,* HFD conditions are often achieved by exposing cultured cells to lipid-rich media. This is typically accomplished by supplementing the media with free fatty acids (FFAs), such as palmitate, oleate, or a combination of these fatty acids, to mimic the cellular environment associated with HFDs (Moliterni et al., 2024). The concentration and preparation of these fatty acids, including their conjugation with carriers like bovine serum albumin (BSA), are carefully controlled to prevent lipotoxicity while maintaining physiological relevance (Ko et al., 2020). Even *in vitro*, the composition of macromolecules is adapted to simulate the metabolic conditions occurring *in vivo*. Short-term studies (24–72 h) focus on acute responses, such as changes in lipid absorption, barrier integrity, and inflammatory signaling, while long-term experiments (lasting several weeks) investigate chronic adaptations, including stem cell proliferation, epithelial integrity, and metabolic dysregulation (Ko et al., 2020; An et al., 2022).

Organoid-based 3D cultures are widely used for their ability to mimic intestinal microarchitecture and cellular dynamics. These systems incorporate Matrigel scaffolds and collagen support to create structural and biochemical environments, allowing assessment of epithelial proliferation, gene expression, and permeability changes (Gori et al., 2020). For example, canine colonoid-derived monolayers have been shown to model gut barrier dysfunction induced by palmitic acid through reductions in TJ proteins like ZO-1 and E-cadherin (Nagao and Ambrosini, 2024). Hydrogel scaffolds and synthetic matrices further enhance the versatility of these models, enabling the incorporation of extracellular matrix (ECM) components and growth factors that support differentiation and metabolic responses (Zeiringer et al., 2023). These systems frequently use Caco-2 and HT29-MTX cells to replicate enterocytes and mucus-producing goblet cells, allowing for studies of permeability and nutrient transport (Nagao and Ambrosini, 2024). Additionally, microfluidic platforms, such as gut-on-chip devices, integrate fluid dynamics to emulate physiological conditions, including nutrient flow and mechanical stress (Zeiringer et al., 2023).

## Effect of HFD on intestinal structure and function

**FIGURE 2:**
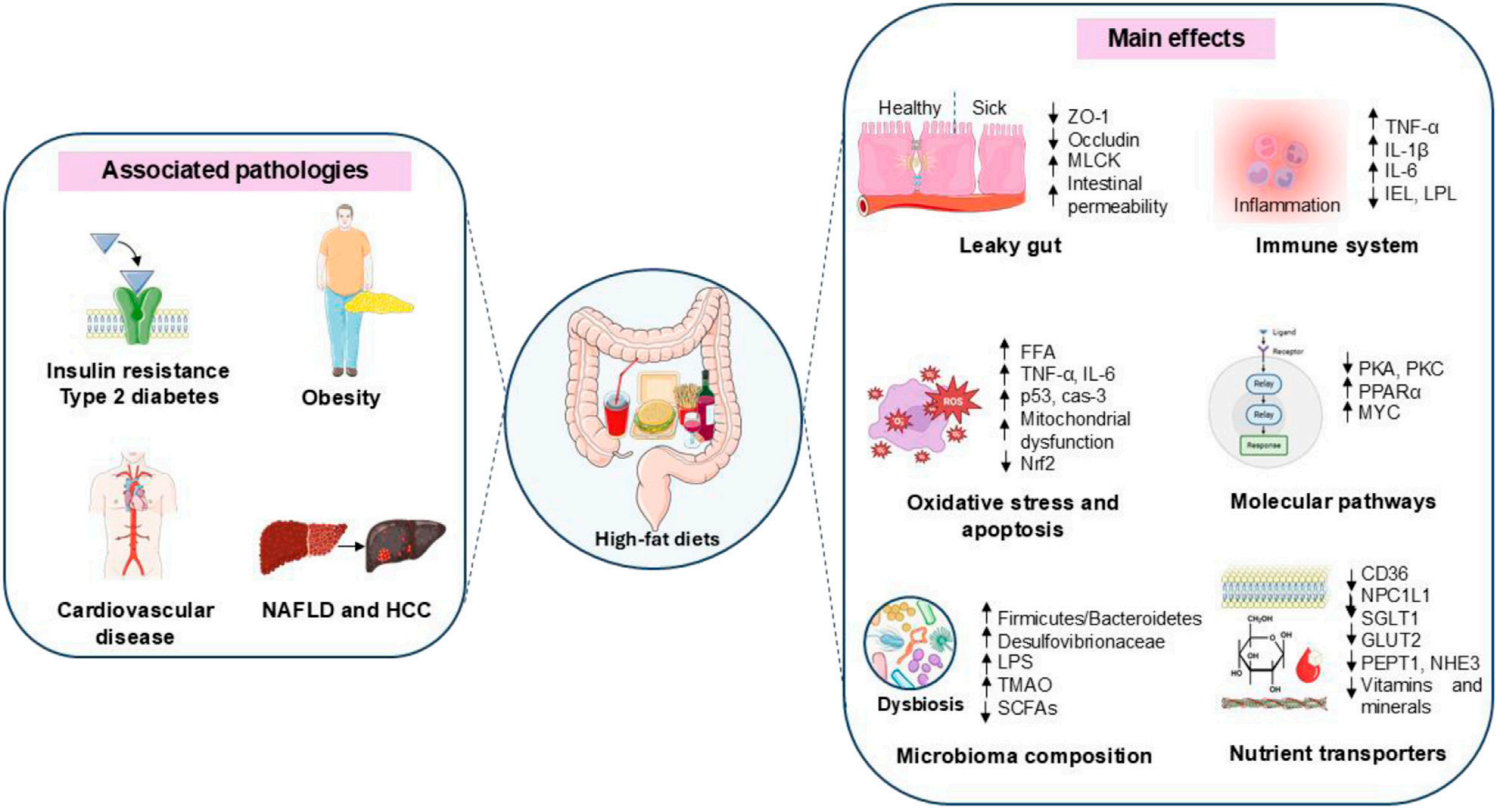
Main effects of HFDs on intestinal structure and metabolism. HFD is associated with several metabolic diseases, including insulin resistance, type 2 diabetes mellitus (T2DM), obesity, cardiovascular disease, NAFLD, and hepatocellular carcinoma (HCC). These effects are mediated through alterations in gut structure and function, leading to increased permeability (leaky gut). The mechanisms include: 1) decreased expression of tight junction proteins, such as ZO-1, occludin, and MLCK; 2) inflammation, characterized by an increased release of inflammatory cytokines and a reduction in IELs and LPLs; 3) dysbiosis, with an increased production of trimethylamine (TMA) and a decreased production of SCFAs; 4) impaired nutrient transport due to low expression of key nutrient transporters, including fatty acid translocase (FAT/CD36), fatty acid transport proteins (FATPs), Niemann-Pick C1-like 1 (NPC1L1), sodium-glucose co-transporter 1 (SGLT1), glucose transporter 2 (GLUT2), and 5) altered function of peptide transporter 1 (PEPT1) and sodium-hydrogen exchanger 3 (NHE3).

The type of dietary fat can significantly influence intestinal structure, with SFAs potentially having detrimental effects, while monounsaturated fatty acids (MUFA) and polyunsaturated fatty acids (PUFA) offer protective benefits (Figure 2). SFAs, commonly found in animal products and processed foods, can disrupt the intestinal barrier, leading to increased permeability and a higher risk of metabolic disorders (Hariri and Thibault, 2010). In contrast, MUFAs, which are present in olive oil and avocados, are generally regarded as beneficial for gut health. They enhance the integrity of the intestinal barrier and support the metabolism of intestinal cells, potentially reducing inflammation and fostering a more balanced gut microbiome (Memmola et al., 2022). PUFAs, particularly omega-3s found in fatty fish and flaxseeds, are recognized for their anti-inflammatory effects. Additionally, these fatty acids improve cell membrane fluidity, which aids in nutrient absorption and helps regulate immune responses (Memmola et al., 2022).

HFD stimulates pro-inflammatory signaling pathways, increasing tumor necrosis factor alpha (TNF-α), IL-1β, and IL-6 levels (Hickey et al., 2023; Di Tommaso et al., 2021). Mice fed an HFD composed of 60% calories from fat, primarily SFA, exhibited reduced levels of ZO-1 and occludin and increased expression of TNF-α and IL-6, which activated myosin light chain kinase (MLCK), leading to TJ disruption and increased permeability (Dang et al., 2023; Rohr et al., 2020). In mouse models of HFD-induced obesity, inflammatory cytokines are associated with a marked increase in intestinal permeability. Instead, anti-inflammatory cytokines like IL-22 enhance gut barrier function by promoting PI3K-mediated intestinal epithelial cell proliferation, supporting wound healing, and reducing fatty acid-induced endoplasmic reticulum stress (Dang et al., 2023; Rohr et al., 2020). Similarly, IL-17 aids gut barrier TJ organization through actin1-mediated occludin-F-actin association. These cytokines, critical for maintaining barrier integrity, are often diminished in HFD-fed subjects, weakening gut defenses against barrier breakdown (Di Tommaso et al., 2021). Moreover, HFD-induced dysbiosis exacerbates intestinal permeability by accelerating damage to TJ proteins in the small intestinal mucosa (Rohr et al., 2020).

Intestinal barrier damage due to inflammation is not limited to local effects but has systemic consequences. Once permeability is increased, luminal toxins and bacterial antigens like lipopolysaccharide (LPS) can enter the bloodstream, leading to endotoxemia (Hickey et al., 2023; Xie et al., 2020). Endotoxemia exacerbates systemic inflammation and is strongly linked to metabolic diseases such as insulin resistance, type 2 diabetes, and obesity. This highlights the broader implications of gut permeability alterations and how an HFD can contribute to metabolic dysregulation (Di Tommaso et al., 2021; Hariri and Thibault, 2010).

Oxidative stress plays a key role in amplifying inflammation and microbial disruptions, impairing intestinal permeability under HFD conditions (Hickey et al., 2023; Di Tommaso et al., 2021). Excess dietary fats, especially SFAs, increase reactive oxygen species (ROS) in epithelial cells, damaging TJ cell structures. In HFD-fed animals, markers like lipid peroxides rise, and antioxidant enzymes are depleted, making epithelial cells more vulnerable to ROS damage (Di Tommaso et al., 2021). The resulting oxidative environment activates pathways like the nuclear factor-κB (NF-κB), which promotes inflammation and further disrupts TJs, worsening intestinal permeability. High oxidative stress can also induce apoptosis, forming gaps in the intestinal lining that allow the passage of harmful substances (Di Tommaso et al., 2021). The transition from a normal fat diet (13% fat) to an HFD (60% fat) in mice caused a shift from carbohydrate to fat metabolism and significantly increased cellular proliferation in intestinal crypts (Enriquez et al., 2022). Single-cell RNA sequencing revealed the activation of stress response pathways, upregulation of lipid metabolism genes, and enhanced lipid absorption capacity within 3 days, highlighting rapid intestinal adaptation to dietary changes (Enriquez et al., 2022). A study analyzed the effects of HFDs in 12-month-old mice, revealing reduced villus length, colon length, and crypt depth, indicative of atrophic and structural dysfunction.

Increased intestinal stem cells (ISCs) enhanced regenerative capacity but predisposed to colorectal cancer (Lefebvre et al., 2024). Exposure to HFD compromises intestinal immunity by reducing intraepithelial lymphocytes (IEL) and lamina propria lymphocytes (LPL) within 1 day, with effects intensifying over 3 weeks (Zeiringer et al., 2023; Tanaka et al., 2020). A recent study revealed a sex-dependent response to 14 weeks of HFD administration in C57BL/6J mice. Both sexes demonstrated HFD-induced gut dysbiosis, but females experienced a more pronounced colonic inflammation, marked by increased expression of TLR4, IL-6, and IL-1β, associated with greater intestinal permeability with altered expression of occludin and claudins (Lefebvre et al., 2024).

FFAs were toxic to intestinal T-cells *in vitro* and *in vivo*; lipase inhibitors partially preserved epithelial integrity, reducing luminal FFA, whereas statins failed to protect against FFA toxicity, confirming damage was mediated primarily through the intestinal lumen (Shi et al., 2019). A model of intestinal barrier obtained with human intestinal cells (Caco-2) treated for 4 days with palmitic acid showed reduced expression of junctional proteins (E-cadherin, ZO-1, occludin, tricellulin) and increasing paracellular permeability (Gori et al., 2020). Palmitic acid also induced an inflammatory response with elevated IL-8 levels, effects not observed with oleic acid (Heimann et al., 2016). Palmitic acid was reported to disrupt gut epithelium homeostasis in colonoid-derived monolayers, reducing ZO-1 expression and Transepithelial-Transendothelial Electrical Resistance (TEER) within 24 h (Nagao and Ambrosini, 2024).

## Molecular mechanisms underlying HFD effects on the intestine

At the molecular level, HFD effects are mediated by different signaling pathways. PPARα, a nuclear receptor activated by dietary lipids, drives crypt expansion and villus elongation but also promotes lipid droplet accumulation, linking structural changes to functional impairments (Stojanović et al., 2021; Karwad et al., 2019). Protein kinases such as PKA and PKC, which regulate nutrient transporter activity, are inhibited under HFD conditions, further exacerbating the malabsorption of lipids, carbohydrates, and proteins (Torelli Hijo et al., 2019). MYC plays a dual role in nutrient absorption and metabolic regulation. While its normal activity supports efficient glucose and lipid absorption, excessive MYC activity, particularly in obesity, exacerbates metabolic dysfunction (Luo et al., 2021).

HFDs significantly disrupt the intricate balance of intestinal hormone signaling and impair the gut-liver axis, both of which are crucial for maintaining energy homeostasis. A recent study registered how MYC disruption promotes glucagon-like peptide-1 (GLP-1) production, a hormone that improves glucose homeostasis by stimulating insulin secretion and enhancing glucose absorption (Little et al., 2007). Under normal conditions, dietary fats in the small intestine stimulate the release of appetite-regulating hormones, including cholecystokinin, GLP-1, and peptide YY (PYY). These hormones work synergistically to slow gastric emptying, reduce hunger, and enhance feelings of satiety. However, chronic exposure to an HFD diminishes these regulatory mechanisms, resulting in faster gastric emptying, blunted hormonal responses, and weakened gut-liver communication. These deleterious effects drive excessive energy intake, weight gain, and dysregulated lipid and glucose metabolism (Hou et al., 2022).

## HFD-induced gut microbiome alteration

The composition and function of gut microbiome are profoundly shaped by dietary patterns, with HFDs emerging as a significant factor in altering microbial dynamics and contributing to systemic health outcomes (Pflughoeft and Versalovic, 2012; de Vos et al., 2022). The relationship between HFDs and microbiomes has been increasingly recognized as a key player in metabolic, inflammatory, and neoplastic diseases (Malesza et al., 2021). HFDs induce notable shifts in the gut microbiota, disrupting homeostasis and leading to dysbiosis (Zsálig et al., 2023). Some diets, such as those rich in SFA from lard and palm oil, are associated with reduced microbial diversity and a pronounced increase in the *Firmicutes/Bacteroidetes* ratio, a hallmark linked to enhanced energy extraction and weight gain (Andújar-Tenorio et al., 2022; Nogal et al., 2021). Additionally, HFDs induce the growth of pro-inflammatory taxa, such as Desulfovibrionaceae, which produce LPS capable of compromising the gut barrier, inducing systemic inflammation *via* toll-like receptors (TLRs) and establishing a pathway to metabolic dysfunction (Zsálig et al., 2023). These changes are associated with decreased levels of beneficial metabolites, such as SCFAs (Malesza et al., 2021; Losacco et al., 2018). In addition, the microbial metabolism of dietary fats leads to an increase in secondary bile acids, which contribute to systemic inflammation and insulin resistance (Zsálig et al., 2023). In contrast, diets rich in MUFAs and PUFAs have a milder effect on microbial diversity and support a more balanced microbiota. Their anti-inflammatory properties reduce pro-inflammatory cytokines while promoting the production of SCFAs by gut bacteria. Butyrate not only provides energy for colonic epithelial cells but also strengthens TJs, improving gut barrier stability and reducing susceptibility to damage caused by dietary fats (Beyaz et al., 2016).

## HFDs disrupt intestinal nutrient transport

Beyond systemic impacts, HFDs exert profound effects on intestinal physiology including crypt elongation, villus shortening, and microvilli disruption. These changes significantly reduce the absorptive surface area, limiting nutrient uptake (Torelli Hijo et al., 2019). Furthermore, high-fat, high-sucrose, low-fiber diets caused intestinal shortening in mice compared to mice fed normal chow (Karwad et al., 2019).

Increased crypt depth is one of the hallmarks of HFD-induced intestinal remodeling. ISC proliferation under HFD conditions results in crypt expansion but at the expense of differentiation into functional absorptive enterocytes. This imbalance results in a reduced number of mature enterocytes, the main cells responsible for efficient nutrient absorption. Paneth cells, essential for maintaining the ISC niche, are also affected by compromising epithelial homeostasis (Beyaz et al., 2016; D’Aquila et al., 2019).

Lipid transporters, including FAT/CD36, FATPs, and NPC1L1, are essential for the transport of long-chain fatty acids and cholesterol, from the lumen into enterocytes. Chronic HFD exposure significantly reduces the expression of these transporters, leading to inefficient lipid uptake (Torelli Hijo et al., 2019). Intracellular lipid droplet accumulation within enterocytes, a hallmark of HFD-fed animals, further disrupts lipid processing and absorption (Karwad et al., 2019; Wiśniewski et al., 2015). Compensatory upregulation of proteins such as liver fatty acid-binding protein (L-FABP) and microsomal triglyceride transfer protein (MTTP) reflects the intestine’s attempt to manage excess lipid intake but exacerbates systemic dyslipidemia in mice long-term fed on an HFD (Petit et al., 2007). Moreover, in a murine model, chronic HFD consumption enhances intestinal lipid absorption by upregulating FATP-4 and MTTP, increasing cell proliferation, and expanding the absorptive area (Jais and Brüning, 2017).

Carbohydrate absorption is mediated by SGLT1 and GLUT2. HFDs reduce the expression of these transporters, impairing glucose absorption (Torelli Hijo et al., 2019; Petit et al., 2007). The effect, coupled with systemic metabolic dysfunctions like insulin resistance, highlights the widespread consequences of intestinal transporter dysregulation (Beyaz et al., 2016).

The absorption of di- and tripeptides is facilitated by PEPT1, while NHE3, an antiporter that contributes to blood buffering capacity, maintains the ionic balance that ensures protein digestion. HFDs impair the functionality of these transporters, leading to reduced protein absorption/digestion and amino acid deficiencies (D’Aquila et al., 2019).

The altered bile acid metabolism consequent to HFD consumption significantly impairs fat-soluble vitamin (A, D, E, and K) absorption. Bile acids are critical for micelle formation, which facilitates the solubilization and uptake of these vitamins. Disruptions in bile acid recycling reduce micelle formation, limiting the bioavailability of these essential micronutrients (Enriquez et al., 2022). Moreover, HFD-associated inflammation induces the downregulation of mineral transporters and compromises the uptake of calcium, magnesium, zinc, and iron, exacerbating deficiencies linked to metabolic syndrome (Kawai et al., 2021; Yoo et al., 2024).

## Systemic impacts of dysbiosis induced by HFDs

The consequences of HFD-induced dysbiosis extend across multiple physiological systems, manifesting most prominently as metabolic disorders. Obesity, a condition strongly associated with HFD consumption, is exacerbated by the enhanced capacity of microbiota to harvest energy from food and the promotion of adipogenesis through altered SCFA profiles (Andújar-Tenorio et al., 2022). Dysbiosis also supports the development of insulin resistance by promoting low-grade systemic inflammation and impairing insulin signaling pathways (Pflughoeft and Versalovic, 2012). Furthermore, increased intestinal barrier permeability, driven by reduced butyrate production and impaired TJ, amplifies the translocation of microbial products such as LPS into the bloodstream, causing metabolic endotoxemia (Malesza et al., 2021). In the liver, altered bile acid metabolism and lipid accumulation, facilitated by intestinal microbial shifts, contribute to NAFLD (Nogal et al., 2021).

HFD is linked to cardiovascular disease partly through the alteration of gut microbiota. In this condition, an increased population of facultative anaerobes, including *Escherichia coli*, enhances the breakdown of dietary choline into TMA. Once absorbed, TMA is oxidized in the liver to form trimethylamine N-oxide (TMAO). Elevated TMAO levels contribute to atherosclerosis, exacerbating vascular plaque formation (Kasper et al., 2021). HFDs also enhance the carcinogenic potential of the gut microbiota through the overproduction of secondary bile acids and the sustained activation of pro-inflammatory pathways, creating a microenvironment conducive to tumorigenesis, particularly in colorectal tissues (de Vos et al., 2022; Malesza et al., 2021).

## HFDs interfere with the endocannabinoid system

The effects of cannabinoids on intestinal metabolism can have both therapeutic and adverse consequences, influencing conditions like IBS, IBD, and obesity (Cravatt et al., 2001). HFDs have been shown to upregulate ECS activity through increased endocannabinoid levels, particularly AEA and 2-AG. Through CB1 and CB2, these endocannabinoids amplify lipogenesis and decrease energy expenditure, contributing to obesity (Iannotti and Vitale, 2021; Pertwee et al., 2010; Lu and Mackie, 2021). Elevated AEA levels are driven by increased activity of NAPE-PLD, the enzyme responsible for endocannabinoid biosynthesis, particularly in adipose tissues. Likewise, HFDs enhance DAGL expression, furthering 2-AG synthesis in white and brown adipose tissues (Mehrpouya-Bahrami et al., 2017).

The dysregulation of ECS components by HFDs is not limited to adipose tissues; alterations are also observed in the liver and brain, contributing to systemic metabolic and inflammatory dysfunctions. The overactivation of CB1 receptors in the hypothalamus under HFD conditions promotes hyperphagia, decreases thermogenesis, and fat deposition in peripheral tissues, exacerbating obesity and its associated comorbidities (Iannotti and Vitale, 2021). A recent study highlights that SR141716A, a CB1 receptor antagonist, mitigates diet-induced obesity by reducing inflammation, improving gut barrier integrity, and modulating the gut microbiome. SR141716A increases beneficial gut bacteria, particularly *Akkermansia muciniphila*, and boosts SCFA production, including propionate and butyrate, which support metabolic and anti-inflammatory processes (Mehrpouya-Bahrami et al., 2017). These effects are independent of caloric restriction, emphasizing the therapeutic potential of CB1 antagonists in managing obesity and metabolic disorders.

Conversely, some studies indicate that CB1 activation exerts protective effects in response to HFDs. Mice consuming HFD, rich in sucrose, showed increased intestinal permeability and reduced levels of 2-AG and related monoacylglycerols in the colonic epithelium. These effects were more pronounced in CB1-deficient mice (Cuddihey et al., 2022b; Wiley and DiPatrizio, 2022). In a mouse model of Western diet-induced obesity, with chronic access to an HFD and a high-sucrose diet, a reduced endocannabinoid level with increased permeability in the large-intestinal epithelium has been reported. Moreover, CB1^−/−^ mice fed on the obesogenic diet experienced decreased expression of TJ proteins and increased expression of inflammatory markers in the large-intestinal epithelium (Cuddihey et al., 2022b; Wiley and DiPatrizio, 2022). In another study, mice fed on an HFD showed increased intestinal permeability restored by activation of the CB1 receptor (Cuddihey et al., 2022b).

The phytocannabinoids CBD and THC have also demonstrated regulatory effects on intestinal permeability and microbiota composition. Indeed, mice fed on an HFD rich in cholesterol experienced gut microbiota disturbances, marked by an elevated *Bacteroidetes/Firmicutes* ratio and an increased abundance of *Mucispirillum schaedleri*, contributing to intestinal inflammation and NAFLD progression. Administration of THC (2.5 mg/kg) and CBD (2.39 mg/kg) reversed these detrimental alterations, with CBD particularly effective at restoring *Firmicutes*, reducing inflammatory microbes, and increasing beneficial bacteria such as *Clostridia* and Ruminococcaceae (Gorelick et al., 2022). These results suggest that CBD can modulate the gut-liver axis and potentially alleviate NAFLD.

In a model of HFD-induced intestinal dysfunction, PEA showed significant protective effects by improving gut barrier integrity and promoting the growth of beneficial microbes like *Bifidobacterium* and *Turicibacter sanguinis*, which supported intestinal homeostasis (Pirozzi et al., 2023). Moreover, PEA reduced intestinal inflammation, enhanced serotonin synthesis, and decreased kynurenine levels by modulating tryptophan metabolism (Schwartz et al., 2008).

Research suggests that HFDs can influence the activity and expression of FAAH and MAGL, potentially altering endocannabinoid tone and its related physiological effects. A study demonstrated that an HFD increased the activity of MAGL in the hypothalamus, a key brain region involved in the regulation of appetite and energy homeostasis. This enhancement of MAGL activity may contribute to the dysregulation of appetite and feeding behaviors observed in obesity (Nomura et al., 1979). Furthermore, HFD-induced obesity has been shown to modulate FAAH expression in various tissues. A study reports that chronic exposure to HFD in mice led to a decrease in FAAH expression in the liver, which could result in higher levels of AEA (DiPatrizio et al., 2011). This could potentially exacerbate the inflammatory response and increase the risk of metabolic diseases, as AEA is involved in both immune modulation and fat metabolism (Ignatowska-Jankowska et al., 2015). Inflammatory mediators, such as cytokines and FFAs, have been shown to regulate the activity of both FAAH and MAGL (Tandon et al., 2021; Sipe et al., 2002). Additionally, HFD-induced changes in the gut microbiota could further influence endocannabinoid signaling by modulating enzyme activity (Ramesh et al., 2013; Kumar Singh et al., 2019).

## Dietary and therapeutic interventions to counteract HFD damage

Therapeutic interventions against HFDs have become a critical area of research due to the increasing prevalence of obesity and metabolic diseases such as type 2 diabetes, NAFLD, and cardiovascular diseases. Studies have explored both non-pharmacological and pharmacological interventions to mitigate these effects.

Dietary modifications are one of the primary approaches, with several studies showing that reducing dietary fat intake, particularly SFA, can significantly improve metabolic outcomes. A randomized trial demonstrated that a Mediterranean diet, which is lower in SFA and higher in MUFA, led to improved insulin sensitivity and a reduction in markers of inflammation in individuals with metabolic syndrome (Papadaki et al., 2020). Furthermore, supplementation with omega-3 fatty acids, known for their anti-inflammatory properties, has shown promising results in both animal and human studies. A study found that omega-3 supplementation attenuated HFD-induced hepatic steatosis and improved lipid profiles in mice, suggesting potential therapeutic benefits (Kuda et al., 2018; Cani et al., 2007).

Given the significant health burden of HFD-induced dysbiosis, strategies to restore microbial balance have garnered considerable attention. Emerging therapeutic strategies include the use of probiotics and prebiotics, which can modulate the gut microbiota and potentially mitigate the inflammatory effects induced by HFDs. A study found that probiotics administration to mice fed on an HFD reduced systemic inflammation and improved insulin sensitivity, suggesting a potential role for gut microbiota modulation in preventing metabolic dysfunction (Davani-Davari et al., 2019). The inclusion of prebiotics, such as inulin and resistant starch, selectively supports the growth of beneficial bacteria, while replacing SFA with unsaturated fats, such as those found in extra virgin olive oil, has shown promise in promoting a healthier microbial profile (Wang et al., 2011; Liu et al., 2021). Dietary interventions focusing on increasing fiber intake have been demonstrated to enhance microbial diversity and SCFA production, counteracting the deleterious effects of HFDs (de Vos et al., 2022; Herman et al., 2022). The supplementation of dietary fiber, omega-3 fatty acids, or prebiotics has shown the potential to mitigate the negative effects of HFDs on gut permeability (An et al., 2022). Fiber supports TJ integrity, while omega-3 fatty acids exhibit anti-inflammatory properties that counteract the effects of SFAs (An et al., 2022). Moreover, certain probiotics, such as *Lactobacillus* and *Bifidobacterium* strains, are also beneficial as they reduce gut inflammation and support TJ maintenance (Di Tommaso et al., 2021). The development of postbiotics, which include bioactive compounds like SCFAs and bacteriocins, provides another promising avenue for harnessing the microbiome’s therapeutic potential without requiring live microbes (Herman et al., 2022).

Pharmacological treatments also play a key role in counteracting the negative effects of HFDs. For instance, metformin, a commonly prescribed drug for type 2 diabetes, improves insulin sensitivity and reduces fat accumulation in the liver. Additionally, newer agents targeting specific metabolic pathways, such as the GLP-1 agonist liraglutide, have been shown to reduce fat mass and improve glycemic control in patients with obesity and type 2 diabetes (Blagov et al., 2023). Fibrates, including fenofibrate, are hypolipidemic drugs that activate PPARα to combat metabolic disorders linked to HFD. A recent study reveals that the activation of PPARα, through synthetic agonists, alleviates palmitic acid-induced lipotoxicity, restoring key cellular processes, such as autophagy and endoplasmic reticulum homeostasis (Mishra et al., 2022).

Considering that chronic inflammation is a hallmark of HFD-induced gut damage, the use of nonsteroidal anti-inflammatory drugs and other anti-inflammatory agents has been studied for their potential to alleviate inflammation and improve intestinal barrier function. For example, mesalazine (a common treatment for IBD) has shown some promise in reducing intestinal inflammation and improving gut permeability in preclinical studies involving HFD-fed animals (Van der Windt et al., 2018). However, long-term use of non-steroidal anti-inflammatory drugs can lead to GI side effects, limiting their therapeutic applicability.

As mentioned above, oxidative stress plays a significant role in gut injury associated with HFDs. Studies have suggested that antioxidants like N-acetylcysteine (NAC) and curcumin can reduce oxidative damage, modulate inflammatory pathways, and improve intestinal permeability (Blagov et al., 2023). NAC, for instance, has shown promise in reducing intestinal inflammation and maintaining the integrity of the epithelial barrier in animal models. However, the clinical translation of these findings remains to be fully explored (Blagov et al., 2023; Knowler et al., 2002).

Exercise is another cornerstone of therapeutic interventions. Regular physical activity helps to counteract the negative effects of an HFD by improving muscle insulin sensitivity and promoting weight loss. A study showed that moderate-intensity exercise significantly reduced the risk of developing type 2 diabetes in individuals with prediabetes, even when they maintained an HFD (Knowler et al., 2002). In animal models, exercise combined with dietary changes has been shown to reduce liver fat content and improve metabolic function (Van der Windt et al., 2018).

Emerging therapeutic approaches, such as fecal microbiota transplantation (FMT), offer innovative solutions for re-establishing microbial equilibrium in dysbiotic individuals. By introducing a diverse and healthy microbial community, FMT has demonstrated efficacy in reversing dysbiosis in conditions such as *Clostridium difficile* infection and potentially HFD-induced metabolic disorders (Pflughoeft and Versalovic, 2012; Andújar-Tenorio et al., 2022).

## Therapeutic potential of OEA in HFD-induced intestinal dysfunction

**FIGURE 3:**
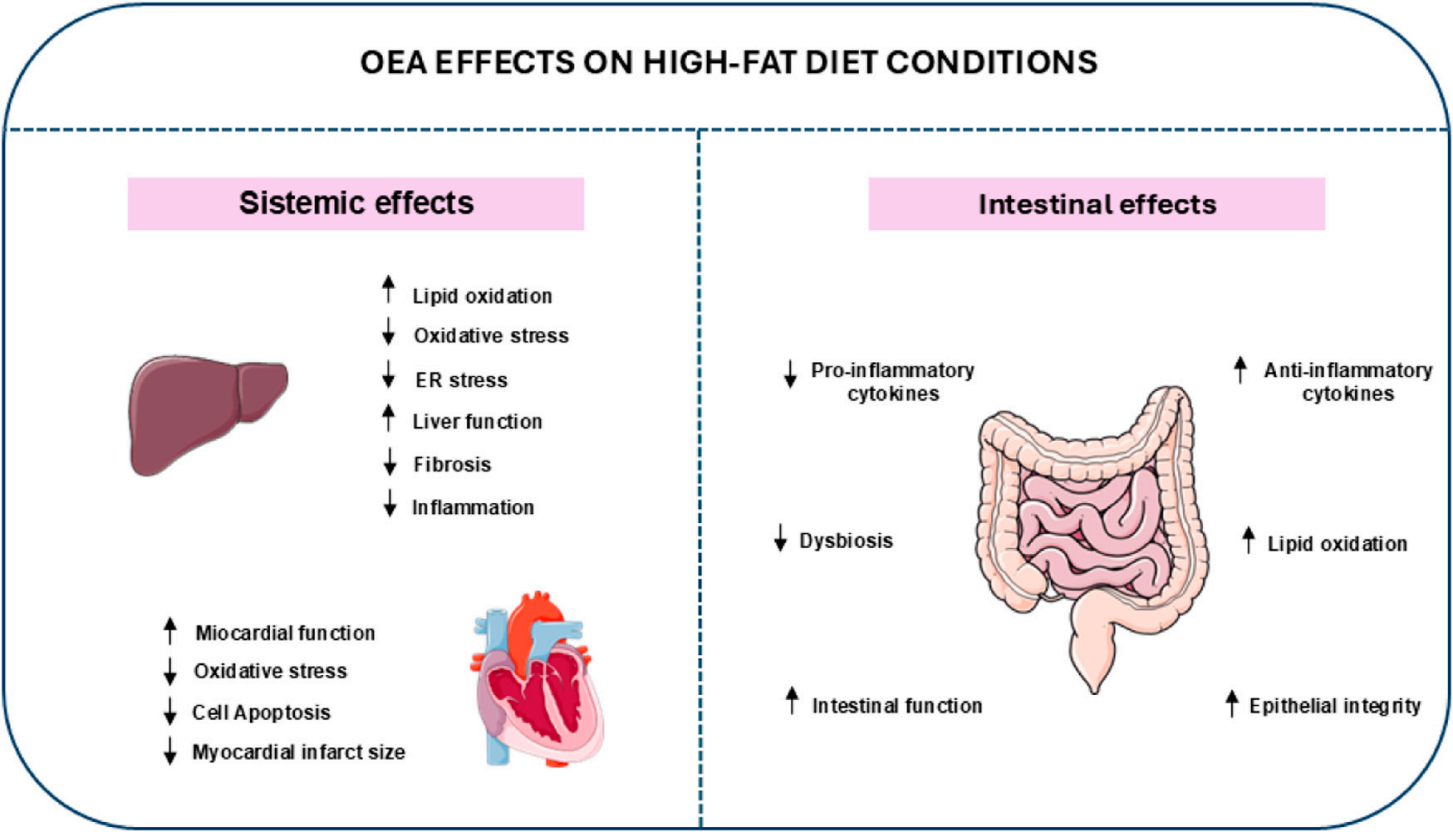
Systemic and intestinal effects of OEA. At a systemic level, OEA has beneficial effects on lipid metabolism, enhances cardiovascular function, and mitigates inflammation. Within the intestinal environment, OEA plays a crucial role in strengthening the integrity of the intestinal barrier, modulating microbiota composition, and regulating local inflammatory responses, all of which contribute to maintaining overall gut health.

OEA positively regulates lipid metabolism by stimulating fatty acid uptake, decreasing lipid accumulation in hepatocytes, and enhancing fatty acid oxidation and lipolysis (Giudetti et al., 2021; Bowen et al., 2017). Moreover, OEA exerts prominent roles in intestinal physiology, contributing to overall gut health (De Filippo et al., 2023) (Figure 3). The intestine, particularly the small intestine, is equipped with PPARα receptors, like other metabolically active tissues such as the liver and skeletal muscle, making this organ highly responsive to OEA (Terrazzino et al., 2004). OEA has been shown as a potential therapy for obesity-related liver disorders like NAFLD (Giudetti et al., 2021). In HFD-fed rats, daily intraperitoneal injection of OEA (10 mg/kg) for 2 weeks reduced liver fat accumulation and improved lipid metabolism by decreasing lipogenic markers and enhancing lipid oxidation through PPARα activation (Giudetti et al., 2021). OEA also reduced oxidative and endoplasmic reticulum stress and improved liver function by lowering markers of oxidative damage and increasing antioxidant enzymes (Giudetti et al., 2021). In an experimental model of mouse liver fibrosis, OEA mitigates liver fibrosis by targeting hepatic stellate cells *via* a PPARα-dependent pathway. OEA reduces fibrosis markers such as α-SMA and collagen, alleviates inflammation, and modulates extracellular matrix remodeling while suppressing TGF-β1 signaling. Notably, these protective effects were absent in PPARα knockout models, highlighting the critical role of PPARα in mediating OEA’s anti-fibrotic actions (Chen et al., 2015). A study reported how the intraperitoneal injection of OEA exerted significant cardioprotective effects in HFD-induced diabetic rats with myocardial ischemia-reperfusion injury. OEA pretreatment reduced creatine kinase, lactate dehydrogenase, and malondialdehyde levels. Additionally, OEA reduced myocardial infarct size, improved myocardial tissue structure, and mitigated cell apoptosis. Through TRPV1 receptors, OEA activated the PI3K/Akt signaling pathway, reducing apoptosis-related caspase-3, and enhancing the anti-apoptotic Bcl-2/Bax ratio (Yao et al., 2022). Intraperitoneal OEA administration in HFD-fed mice reversed dopamine deficiency induced by the diet in a PPARα-dependent manner (Tellez et al., 1979). A recent study, in mice long-term (12 weeks) treated with HFD, has introduced an “intestinal OEA factory” designed for the controlled *in situ* release of OEA. This was achieved by engineering *Lactobacillus paracasei* F19 (LP) to express the human NAPE gene, enabling the production of OEA in response to a dietary supply of ultra-low oleate. This innovative system resulted in a significant weight reduction and improved metabolic dysfunction. Additionally, there was a notable improvement in depressive and anxiety-like behaviors, which correlated with the restoration of duodenal barrier function, the reestablishment of the *Firmicutes/Bacteroidetes* ratio, and an increase in beneficial bacteria, including *Lactobacillus*, *Prevotella,* and *Parabacteroides* (Seguella et al., 2025). OEA exhibits potent anti-inflammatory effects by reducing levels of pro-inflammatory cytokines such as TNF-α and IL-6 in HFD feeding. It simultaneously enhances anti-inflammatory markers like IL-10, promoting a shift toward a balanced immune response (De Filippo et al., 2023). OEA has demonstrated the ability to increase lipid oxidation, specifically in the jejunum of HFD-fed C57BL/6J mice, by increasing PPARα, FAT/CD36, and FATP1 expression. Similar effects were observed in the liver and duodenum but not in the ileum. This action prevents excessive lipid accumulation in different tissues, thereby improving metabolic parameters like insulin sensitivity and lipid profiles (Fu et al., 2007).

OEA promotes a shift in mice microbiota composition toward a “lean-like” profile, characterized by increased *Bacteroidetes* and reduced *Firmicutes* populations. These changes are accompanied by decreased expression of pro-inflammatory cytokines in Peyer’s patches, underscoring the dual role of OEA in microbiota modulation and immune homeostasis (Di Paola et al., 2018).

OEA has been shown to restore epithelial integrity *in vitro* by modulating PPARα and TRPV1 receptor pathways (Karwad et al., 2017b). A study using intestinal Caco-2 cells demonstrated that OEA prevents the hypoxia-induced reduction in TEER, a measure of barrier integrity, and maintains TJ function. By stabilizing TEER, OEA prevents the paracellular passage of macromolecules and harmful substances, including LPS (Karwad et al., 2019).

A pilot study in healthy young adult men demonstrated that an oral supplement containing spermidine, nicotinamide, PEA, and OEA decreased TNF-α and ROS in stimulated macrophages (Rhodes et al., 2024). Moreover, a double-blind placebo-controlled study demonstrated that 2 months’ supplementation with a complex of epigallocatechin-3-gallate with NAPE ameliorated oxidative stress-related markers of overweight and class I obese subjects (Cazzola and Rondanelli, 2020).

## Conclusion

In conclusion, the interplay between HFDs and the ECS plays a critical role in the development of metabolic disorders, including obesity and intestinal dysfunction. HFDs enhance the activity of the ECS by increasing the levels of endocannabinoids such as AEA and 2-AG, which affect various metabolic pathways, including lipid metabolism and energy balance. This dysregulation contributes to systemic metabolic and inflammatory dysfunctions, exacerbating obesity and related comorbidities (Piomelli, 2003). While cannabinoids like THC and CBD show potential for therapeutic applications in modulating gut permeability and microbiota composition, their effects can vary depending on the context and the specific receptors they target. Compounds such as OEA, which work through alternative pathways such as PPARα activation, offer promising therapeutic prospects due to their ability to improve intestinal barrier integrity, reduce inflammation, and support metabolic health without the adverse effects of traditional ECS-targeted agents (Diao et al., 2022). OEA is a promising therapeutic agent for addressing the harmful consequences of HFDs by simultaneously targeting appetite regulation, gut health, inflammation, and metabolic processes. OEA has the potential to treat metabolic syndrome and related disorders. Together, these findings underscore the complex role of the ECS in mediating the effects of HFDs and highlight the potential of targeting specific ECS components, including OEA, to develop novel therapies for obesity, intestinal dysfunction, and related metabolic disorders. Future investigations should focus on determining optimal dosing strategies and exploring synergistic effects with other treatments.

Despite advancements in research, there are still several gaps that have not been fully addressed. While cannabinoid receptors (CB1 and CB2) and their endogenous ligands have been identified, the precise mechanisms through which they interact with other cellular signaling pathways are not fully understood. Moreover, the processes involved in the synthesis and degradation of endocannabinoids are not fully characterized. Also, it must be considered that significant differences may occur in responses to the endocannabinoid system among individuals, influenced by sex, genetic, environmental, and lifestyle factors (Meccariello et al., 2020; Mohammad et al., 2024). Many studies have focused on specific endocannabinoid compounds without exploring their interactions with other dietary components or the gut microbiome, which may influence their pharmacological effects (Cani et al., 2016). In addition, it should be noted that some endocannabinoids, such as 2-AG, represent a source of arachidonic acid for prostaglandin synthesis (Nomura et al., 2011). Thus, the administration of endocannabinoids, or strategies to increase their synthesis, may have far-reaching effects that go beyond the CB1 and CB2 receptors. Another point that is to be considered is the lack of standardized methodologies across studies, leading to variability in results and conclusions (Gouvêa-Silva et al., 2023). Several studies have utilized small sample sizes or animal models that may not fully represent human physiology, limiting the applicability of their findings to clinical settings. Also, a few studies have attempted to translate preclinical findings into human trials, highlighting a significant gap in our understanding of the therapeutic potential of endocannabinoid compounds.

To address these gaps, future research needs to adopt more comprehensive approaches, including larger, well-controlled clinical trials that consider the multifactorial nature of diet and metabolism. Research on drugs that modulate the ECS is ongoing, but there are still significant challenges in creating effective medications that are free from side effects. More clinical trials are needed to establish the efficacy and safety of endocannabinoid-based treatments for various conditions. This includes understanding optimal dosing, delivery methods, and potential side effects. This could provide a clearer understanding of the role of endocannabinoids in intestinal health. In the future, more mechanistic studies are warranted to elucidate the detailed mechanisms of action of endocannabinoids in the gut. In addition, better-designed clinical trials are needed to explore the full therapeutic potential of endocannabinoids on and through the gut.
